# Case Report: Efficacy of ensartinib treatment in pulmonary inflammatory myofibroblastic tumor with a rare *GCC2-ALK* fusion

**DOI:** 10.3389/fonc.2022.934887

**Published:** 2022-08-08

**Authors:** Wenguang He, Xiao Ji, Congcong Song, Shanshan Song, Lixia Liu

**Affiliations:** ^1^ Traditional Chinese Medicine Department, Shanxi Province Cancer Hospital, Shanxi Hospital Affiliated to Cancer Hospital Chinese Academy of Medical Sciences, Cancer Hospital Affiliated to Shanxi Medical University, Taiyuan, China; ^2^ Department of Translational Medicine, YuceBio Technology Co., Ltd., Shenzhen, China

**Keywords:** inflammatory myofibroblastic tumors, *GCC2-ALK*, ensartinib, case report, partial response

## Abstract

**Background:**

Inflammatory myofibroblastic tumors (IMTs) are rare with distal metastasis. Approximately 50% of patients have anaplastic lymphoma kinase (ALK) fusion. Patients with non-small cell lung cancer with ALK fusion are usually highly sensitive to ALK tyrosine kinase inhibitors (TKIs), but the application of TKI in IMT needs further exploration.

**Case presentation:**

A 66-year-old man was diagnosed with IMT with bone metastasis, cT4N0M1c, IVB stage. Immunohistochemistry results showed that he was ALK positive, and next-generation sequencing revealed *GCC2-ALK* fusion in the IMT. The patient was administered first-line ensartinib 225-mg QD, which targeted *GCC2-ALK* fusion, and denosumab 120-mg Q4w anti-bone metastasis therapy. The patient developed a grade III rash, and the ensartinib dose was reduced to 125 mg QD; consequently, he achieved a partial response (PR), and the side effects significantly reduced. Computed tomography results showed that the patient maintained PR after 7 months of follow-up, and he was still in a state of progression-free survival without obvious side effects after 11 months of follow-up.

**Conclusion:**

To our knowledge, this is the first case of the *GCC2-ALK* fusion type in IMT and the first report showing that the use of ensartinib as a TKI in IMT has clinical benefits.

## Introduction

An inflammatory myofibroblastic tumor (IMT) is a rare tumor; histopathologically, it is composed of differentiated myofibroblast spindle cells and inflammatory cells. IMT is usually found in the lung, retroperitoneum, or abdominal basin, with most cases occurring in children or young people; patients with IMT rarely have distal metastasis (1, 2). Chromosome rearrangement is often found in IMT patients, with *ALK* fusion present in approximately 50% of the cases (3). There are many methods of detecting ALK fusion, such as immunohistochemistry (IHC), fluorescence *in situ* hybridization (FISH), reverse transcription–polymerase chain reaction (RT-PCR), and next-generation sequencing (NGS) detection of DNA or RNA. In this case, ALK fusion was detected in the patient using NGS technology at the DNA level and IHC technology.

Few studies have investigated *ALK* fusion treatment for pulmonary IMT with distal metastasis; such patients respond to tyrosine kinase inhibitors (TKIs) (1, 4). Ensartinib is a new oral, second-generation drug, which is a strong and highly selective ALK-TKI (5, 6). We present the first report of a case of pulmonary inflammatory myofibroblastoma with new *GCC2-ALK* fusion, wherein more than 11 months of clinical benefit was achieved after ensartinib treatment.

## Case presentation

**Figure 1:**
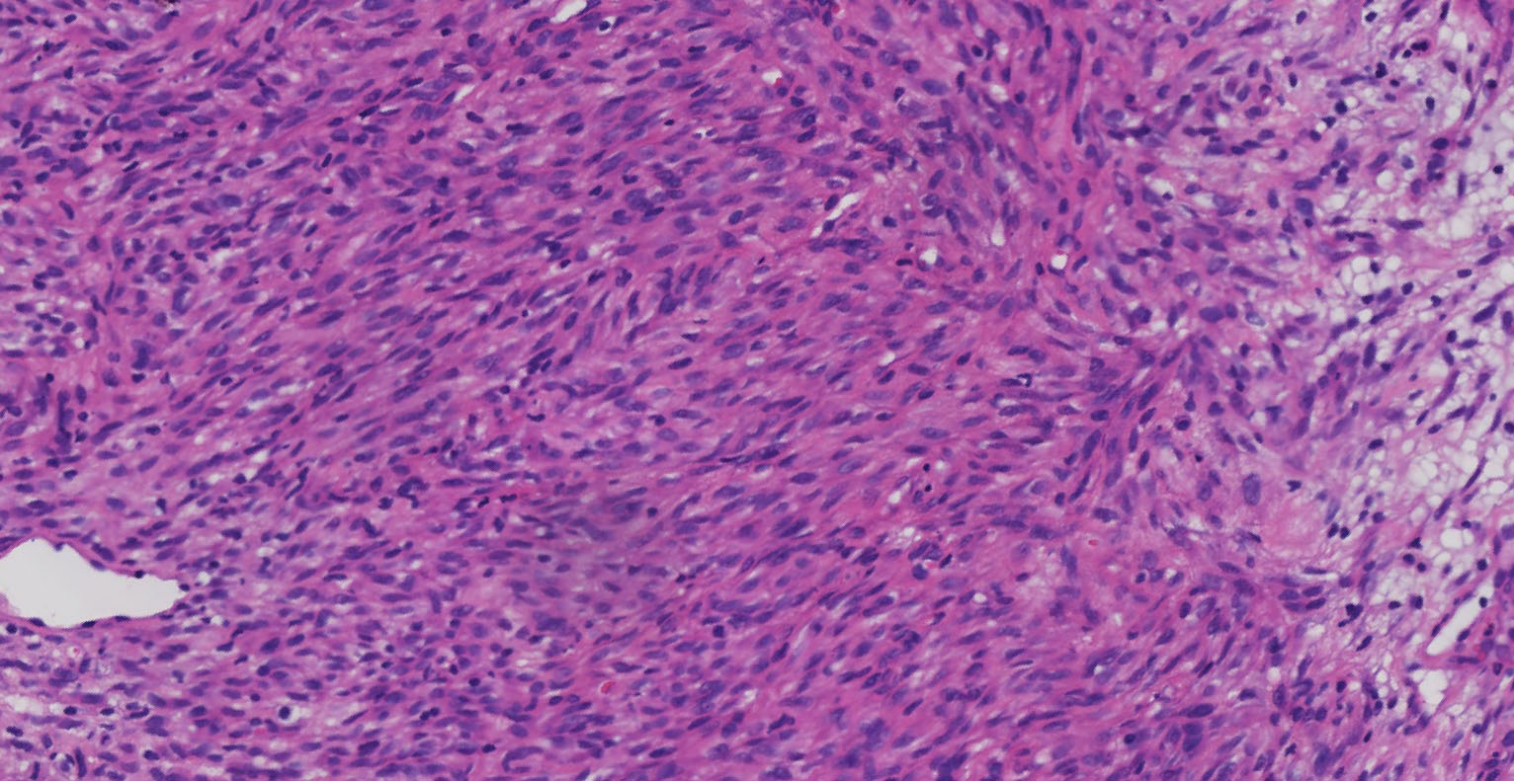
Histology sections from biopsy samples showed a spindle cell infiltrate with accompanying inflammatory stromal cell response (hematoxylin and eosin, original magnification ×200).

**Figure 2:**
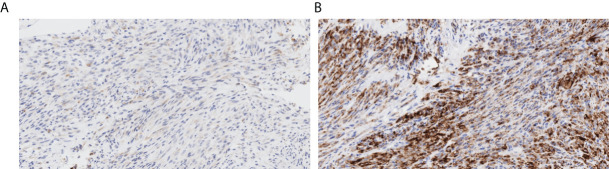
Immunohistochemical findings (original magnification ×200). **(A)** Focal focus weak positive CK7; **(B)** strong ALK expression.

**Figure 3:**
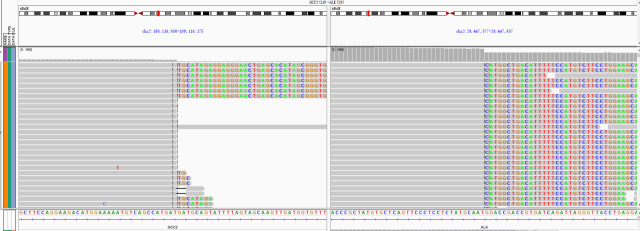
*GCC2-ALK* fusion was identified by next-generation sequencing.

A 66-year-old Chinese man experienced a cough and blood-tinged sputum for >5 months. On admission, the patient had an intermittent cough, a small amount of blood in sputum, a shortness of breath after activity, and mild pain in the left iliac region but no other complaints of obvious discomfort or disease history. He was generally in good health; the performance status (PS) score was 1, and the pain numerical rating scale (NRS) score was 1. The admission examination results of the tumor markers CEA (Carcinoembryonic antigen), CA19-9 (Carbohydrate antigen199), SCC (Squamous cell carcinoma antigen), NSE (Neuron-specific enolase), CYFRA21-1 (Cytokeratin 19 fragment), ProGRP(Progastrin releasing peptide), CA125 (Carbohydrate antigen 125), TPS (Tissue Polypeptide Specific Antigen) and Hsp90α (Heat Shock Protein 90 α) were negative, and the routine blood test results and the main indexes of liver and kidney function were basically normal. Admission positron emission tomography/computed tomography (PET/CT) showed signs of right lung upper lobe cancer and right ischium metastasis and high-density nodules in the right 8th posterior rib, 11th posterior rib, and left iliac bone; metastasis was considered. Tracheoscopy showed the orifice of the upper lobe of the right lung to be almost completely blocked by cauliflower-like tumors, with numerous dirty, white, and dead objects, which bled easily when touched. The biopsy of the upper lobe of the right lung revealed spindle cells arranged in a bundle and mat pattern, scattered lymphocyte infiltration, loose, and edema in some areas (**Figure 1**). Considering the immunohistochemical results: AE1/AE3 (-), vimentin (+), BCL-2 (-), CD34 (-), Ki67 (approximately 60% +), S-100 (-), SMA (-), TTF-1 (-), P63 (weak +), SATB2 (+), STAT6 (-), CK7 (focal weak +), ALK (D5F3) (+), and PD-L1 negative (22C3: TPS<1%) (**Figure 2**), which were consistent with those of malignant tumors, and a patient’s pathological morphology, we suspected pulmonary mesenchymal tumor and established the diagnosis of right pulmonary inflammatory myofibroblastic tumor with bone metastasis. cT4N0M1c, the IVB stage, and the PS score was 1. DNA next-generation sequencing was carried out by using the hybridization capture method with the patient’s tissue samples. We detected 556 cancer-related genes; the average sequencing depth of the target gene detection area was 2697X, and the sequencing depth was sufficient. The sequencing results showed that there was *GCC2-ALK* (G19:A20) fusion (**Figure 3**), and the mutation abundance was 7.11%. No other meaningful target mutations recommended for clinical medication were detected.

**Figure 4:**
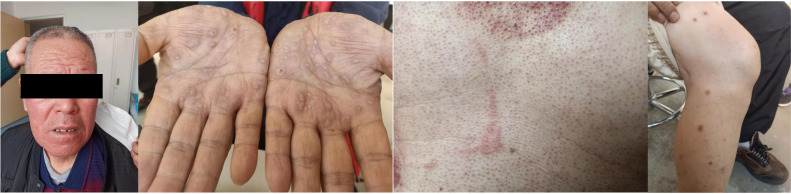
Rash after targeted therapy.

**Figure 5:**
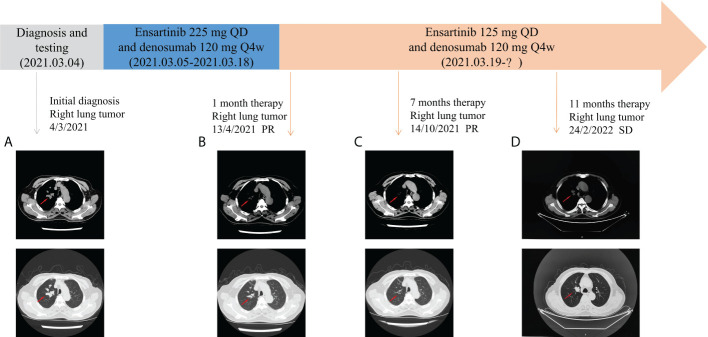
Comparison of CT images before and after treatment with ensartinib. **(A)** Before ensartinib treatment; **(B)** 1 month after ensartinib treatment; **(C)** 7 months after ensartinib treatment; and **(D)** 11 months after ensartinib treatment.

Based on the superior efficacy of ensartinib to crizotinib (6), the patient was started on ensartinib 225- mg QD, which targeted *GCC2-ALK* fusion, and denosumab 120-mg Q4w anti-bone metastasis. After 1 week, the patient reported significant improvement in cough and the disappearance of blood-tinged sputum. However, after approximately 2 weeks of treatment, the patient developed a grade III rash (according to NCI-CTCAE standard version 5.0) appearing first on the face and gradually involving the trunk and bilateral upper and lower limbs (**Figure 4**), with serious pruritus. Therefore, the ensartinib dose was reduced to 125-mg QD. After 2 weeks of drug reduction, the rash on the face and limbs gradually disappeared. In addition, the patient experienced mild loss of taste and constipation during medication , no other discomfort. The patient continued to take the medicine at the stated dose. One month after treatment, the cough significantly improved, there was no blood in sputum, and pain in the left iliac region was relieved. The reexamination of chest CT showed that the focus volume was significantly reduced. According to RECIST 1.1, the better response evaluation was evaluated as partial response (PR). Follow-up chest CT revealed that the patient maintained PR after 7 months. After 11 months of follow-up, chest CT showed that the main focus of the lung tumor was slightly advanced, but the sum of the maximum diameter of the main focus of the tumor did not increase by 20%, and an evaluation of stable disease (SD) was achieved (**Figure 5**). In a whole-body bone scan, the metabolism of T6–T8 and T11, the eighth rib of the right rear was slightly increased, and the right ischium and left iliac bone were changed. A further check was suggested to exclude malignant tumor. The bone metastasis was significantly relieved, and there was no bone pain. After several follow-up visits, the patient’s symptoms were stable, with a small amount of blood in sputum intermittently, which was relieved after symptomatic treatment. There were no major complaints of discomfort and no other drug-related side effects, and the routine blood routine examination and liver and kidney function were normal every time, suggesting the good clinical efficacy of ensartinib. The targeted therapy of ensartinib was continued, and regular outpatient follow-up continued.

## Discussion

Surgery is the main treatment for IMT, while the drug treatment for patients with unresectable tumors is very limited, and the curative effect is low (7, 8). We reported a case of a patient with IMT with bone metastasis who received *GCC2-ALK* fusion and achieved clinical benefits after treatment with ensartinib. There are few reports on TKI treatment for IMT with bone metastasis, with no consistent prediction on the efficacy of TKI in metastatic IMT with *ALK* fusion. A study reported a 32-year-old man with pulmonary IMT with distal metastasis with *TPM3-ALK* fusion, the patient received PR after treatment with crizotinib, the disease progressed after 8 months; then, the patient was treated with ceritinib after drug resistance, after 6 months of treatment, the patient underwent surgical resection and ceritinib adjuvant treatment until IMT relapsed after 18 months (2). *TPM4-ALK* fusion was found in a 40-year-old man with pulmonary IMT with distal metastasis; after chemotherapy and prednisolone had no significant effect, lorlatinib was given and the patient achieved stable disease for 6 months (9). An 8-year-old girl was diagnosed with a highly invasive and metastatic pulmonary IMT after umbilical cord blood transplantation; she was treated with crizotinib to obtain PR but later developed fatal pulmonary toxicity due to diffuse alveolar injury, probably due to a toxic reaction caused by crizotinib (10). The present case was a rare case of *ALK* fusion of pulmonary IMT with distal metastasis, with good clinical benefits after first-line ensartinib treatment. PR was achieved after 1 month of follow-up and lasted for more than 6 months. SD was obtained after 11 months of follow-up. At the time of the submission of the manuscript, the patient had no obvious side effects.

The EORTC trial 90101 CREATE clinical trial included *ALK*-positive and *ALK-*negative patients with advanced/metastatic IMT who received 250 mg of oral crizotinib twice a day; the median PFS of *ALK-*positive and *ALK-*negative patients was 18.0 months (95% CI 4.0-NE) and 14.3 months (95% CI 1.2–31.1), respectively (11). The PFS in our case was more than 11 months; however, long-term benefits still need to be observed. Different primary tumor sites of IMT have different responses to ALK inhibitors; for instance, abdominal organ involvement can benefit better than lung organ involvement (8). Studies have reported that patients with *TP53* mutations have shorter PFS and OS after treatment with ALK-TKI; the TP53 pathogenicity classification that affects the efficacy is mainly the loss of function or like the loss of function (12). In this case, TP53 p.Pro72Arg, a polymorphic site, was detected in the patient; we speculate that it will not have an impact on ALK TKI treatment. This case has shown good clinical efficacy, and the report will enrich the research on the treatment of *ALK-*positive inflammatory myofibroblastic tumors with TKIs.

The *ALK* gene partners reported in IMT are *TPM3, TPM4, CLTC, RANBP2, SEC31L1, EML4, LMNA, TFG, NF1, PRKAR1A, THBS1, TNS1, NUMA1, LRRFIP1, DCTN1, CARS, KIF5B*, and *ATIC* (3, 8, 13–18). Different ALK fusion detection technologies have their own advantages and disadvantages. IHC has the characteristics of a low-cost and fast-detection cycle; compared with molecular methods, it is easier to establish in the diagnostic laboratory, but it can only perform protein level detection and cannot distinguish molecular chaperones. FISH can detect large structural variations at the DNA level and is widely used in clinical laboratories to detect carcinogenic fusion, its advantage is that it requires a small amount of tissues and can detect fusion in cells of interest, while its disadvantage is that it can only detect one gene partner at a time, and it is impossible to determine whether abnormal signals actually lead to the production of fusion transcripts or proteins. RT-PCR has the advantages of low cost and high sensitivity and specificity; however, each fusion needs to be detected separately, and only known fusion types can be detected. DNA based NGS can detect unknown *ALK* fusion partners and evaluate the somatic mutation status in addition to gene fusion; however, similar to FISH, some fusions found by DNA-based NGS may have no functional consequences and may represent a non-functional event. RNA-based NGS is a mature mRNA that is sequenced; fusion detection at the RNA level proves that these fusions are indeed transcribed, and more fusions are detected than with DNA-level detection, however, the most important disadvantage is that RNA is not as stable as DNA, and the quality of RNA does not always meet the requirements of sequencing, especially for FFPE samples. The fusion type of the ALK gene can be preliminarily detected by using probes for DNA-based NGS; RNA and protein-level analysis may be the key to verifying the complex ALK rearrangement function in clinical practice to make the best treatment decision (19–21). In this case, *GCC2-ALK* fusion was detected by DNA-based NGS technology, and the results of the IHC profile showed ALK (+), which further verified the protein function at the protein level, providing theoretical guidance for the use of ensartinib. The reports of *GCC2-ALK* fusion are few, with only a few cases of non-small cell lung cancers (22–26). We also studied the MSK-IMPACT cohort study; the mutation types of IMT in this study are mainly SNV and indel. Only the *RANBP2-ALK* fusion type was detected in IMT gene fusion; at the same time, we saw that *GCC2-ALK* fusion in the MSK-IMPACT study was detected in only one patient, who had lung adenocarcinoma (27). Therefore, there is no report of *GCC2-ALK* fusion in IMT. Studies have shown that NSCLC patients with *GCC2-ALK* fusion have uncertain clinical benefits when using crizotinib; after drug resistance, they may show dissatisfactory responses to the second- or third-generation TKI-targeted therapy, warranting the exploration of more suitable *GCC2-ALK*-targeted drugs (22–24).

Ensartinib is a second-generation ALK inhibitor independently developed in China; the clinical trial of eXalt 3 shows that the efficacy of ensartinib in the first-line treatment of *ALK*-positive patients with advanced NSCLC is significantly better than that of crizotinib, in the intent-to-treat (ITT) population. The mPFS of the ensartinib group was significantly longer than that of the crizotinib group (25.8 vs 12.7 months; hazard ratio, 0.51 [95% CI, 0.35–0.72]; log-rank P<.001), and the treatment was well tolerated (6). However, there is no relevant report on the efficacy of ensartinib in IMT. In this case, the side effects of ensartinib dose reduction to 125-mg QD reduced after 2 weeks, and the clinical benefits remained for ≥11 months, suggesting that appropriately reducing the dose of ensartinib is beneficial to a patient’s prognosis. This case suggests that the *GCC2-ALK* fusion type is the target of ensartinib in IMT treatment; however, its specific therapeutic efficacy can be established through large-scale clinical trials.

## Conclusion

This was a case report on a 66-year-old man with pulmonary inflammatory myofibroblastic tumor with bone metastasis, cT4N0M1c, IVB stage. IHC results combined with NGS showed a new *GCC2-ALK* fusion in IMT. This is the first report of the use of the second-generation ALK-TKI ensartinib in a patient with IMT. The clinical benefit to the patient lasted for more than 11 months, with no obvious side effects. This case suggests that *GCC2-ALK* can be used as a new target for IMT TKI treatment, with ensartinib as a beneficial drug.

## Data availability statement

The original contributions presented in the study are included in the article/supplementary material. Further inquiries can be directed to the corresponding author.

## Ethics statement

Written informed consent was obtained from the individual(s) for the publication of any potentially identifiable images or data included in this article.

## Author contributions

WH and LL took care of the patient and wrote the primary draft. CS edited the draft and incorporated relevant literature, XJ made the graph, and SS participated in draft editing. All authors read and approved the final manuscript.

## Conflict of interest

Authors CS and SS were employed by YuceBio Technology Co., Ltd., Shenzhen, China.

The remaining authors declare that the research was conducted in the absence of any commercial or financial relationships that could be construed as a potential conflict of interest.

## Publisher’s note

All claims expressed in this article are solely those of the authors and do not necessarily represent those of their affiliated organizations, or those of the publisher, the editors and the reviewers. Any product that may be evaluated in this article, or claim that may be made by its manufacturer, is not guaranteed or endorsed by the publisher.
